# A multigenerational study on phenotypic consequences of the most common causal variant of HNF1A-MODY

**DOI:** 10.1007/s00125-021-05631-z

**Published:** 2021-12-24

**Authors:** Jarno L. T. Kettunen, Elina Rantala, Om P. Dwivedi, Bo Isomaa, Leena Sarelin, Paula Kokko, Liisa Hakaste, Päivi J. Miettinen, Leif C. Groop, Tiinamaija Tuomi

**Affiliations:** 1 Folkhälsan Research Center, Helsinki, Finland; 2 Institute for Molecular Medicine Finland (FIMM), University of Helsinki, Helsinki, Finland; 3 Department of Endocrinology, Abdominal Center, Helsinki University Hospital, Helsinki, Finland; 4 Research Programs Unit, Clinical and Molecular Metabolism, University of Helsinki, Helsinki, Finland; 5 Vantaa Healthcare Center, Vantaa, Finland; 6 New Children’s Hospital, Pediatric Research Center, University of Helsinki and Helsinki University Hospital, Helsinki, Finland; 7 Research Programs Unit, Molecular Neurology, and Biomedicum Stem Cell Center, University of Helsinki, Helsinki, Finland; 8 Lund University Diabetes Center, Department of Clinical Sciences Malmö, Lund University, Malmö, Sweden

**Keywords:** Age at onset, Glucagon, HNF1A-MODY, Insulin deficiency, Lipolysis, Maturity-onset diabetes of the young (MODY), MODY3, Monogenic diabetes, NEFA, Polygenic risk score for type 2 diabetes

## Abstract

**Aims/hypothesis:**

Systematic studies on the phenotypic consequences of variants causal of HNF1A-MODY are rare. Our aim was to assess the phenotype of carriers of a single *HNF1A* variant and genetic and clinical factors affecting the clinical spectrum.

**Methods:**

We conducted a family-based multigenerational study by comparing heterozygous carriers of the *HNF1A* p.(Gly292fs) variant with the non-carrier relatives irrespective of diabetes status. During more than two decades, 145 carriers and 131 non-carriers from 12 families participated in the study, and 208 underwent an OGTT at least once. We assessed the polygenic risk score for type 2 diabetes, age at onset of diabetes and measures of body composition, as well as plasma glucose, serum insulin, proinsulin, C-peptide, glucagon and NEFA response during the OGTT.

**Results:**

Half of the carriers remained free of diabetes at 23 years, one-third at 33 years and 13% even at 50 years. The median age at diagnosis was 21 years (IQR 17–35). We could not identify clinical factors affecting the age at conversion; sex, BMI, insulin sensitivity or parental carrier status had no significant effect. However, for 1 SD unit increase of a polygenic risk score for type 2 diabetes, the predicted age at diagnosis decreased by 3.2 years. During the OGTT, the carriers had higher levels of plasma glucose and lower levels of serum insulin and C-peptide than the non-carriers. The carriers were also leaner than the non-carriers (by 5.0 kg, *p*=0.012, and by 2.1 kg/m^2^ units of BMI, *p*=2.2 × 10^−4^, using the first adult measurements) and, possibly as a result of insulin deficiency, demonstrated higher lipolytic activity (with medians of NEFA at fasting 621 vs 441 μmol/l, *p*=0.0039; at 120 min during an OGTT 117 vs 64 μmol/l, *p*=3.1 × 10^−5^).

**Conclusions/interpretation:**

The most common causal variant of HNF1A-MODY, p.(Gly292fs), presents not only with hyperglycaemia and insulin deficiency, but also with increased lipolysis and markedly lower adult BMI. Serum insulin was more discriminative than C-peptide between carriers and non-carriers. A considerable proportion of carriers develop diabetes after young adulthood. Even among individuals with a monogenic form of diabetes, polygenic risk of diabetes modifies the age at onset of diabetes.

**Graphical abstract:**

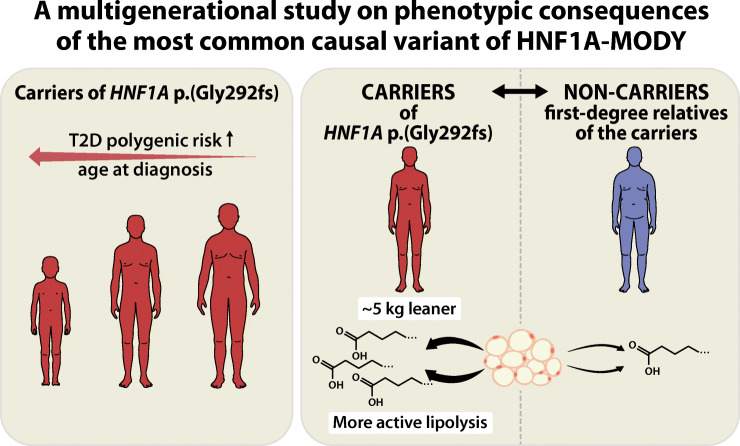

**Supplementary Information:**

The online version contains peer-reviewed but unedited supplementary material available at 10.1007/s00125-021-05631-z.

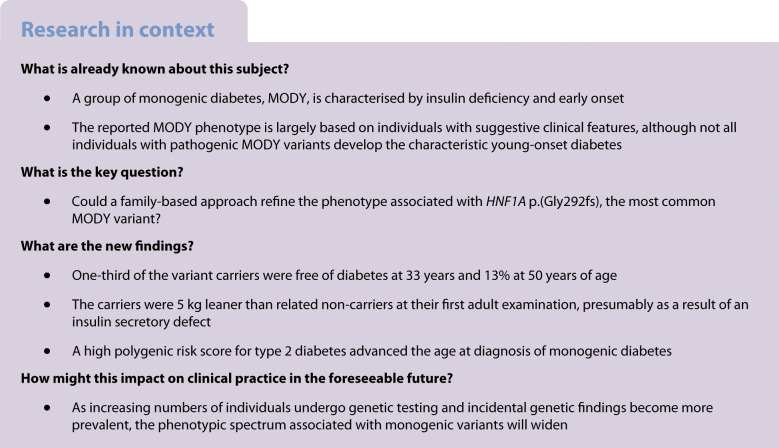



## Introduction

Subtypes of monogenic diabetes called MODY result from rare single-gene variants (reviewed in [1, 2]). MODY was initially defined as an autosomal dominant young-onset non-obese form of non-insulin-dependent diabetes [3, 4]. These features determining patient selection for the first gene discovery studies [5–8] still guide diagnostic testing [9, 10]. However, the phenotype-based selection bypasses the heterogeneous presentation of MODY [9, 11–13], and the reported gene–disease association can be subject to ascertainment bias in at least two ways. First, carriers of pathogenic MODY variants without diabetes or typical phenotype have often not been included in studies. Second, comparing carriers only with patients with polygenic forms of diabetes can lead to misinterpretations regarding the monogenic variant. For example, the level of insulin sensitivity in MODY caused by variants in *HNF1A* (HNF1A-MODY) depends on the comparator group and their genetic background, perhaps irrespective of *HNF1A* altogether [14–16]. And although individuals with HNF1A-MODY are more sensitive to sulfonylureas than those with type 2 diabetes [17], their beta cell response to sulfonylureas is similar to that in control individuals without diabetes [15, 18], rendering it unlikely that the MODY variant directly affects sulfonylurea sensitivity.

Therefore, we conducted a family-based study to systematically characterise the phenotype of carriers of *HNF1A* p.(Gly292fs), the most common causal variant of HNF1A-MODY worldwide, by comparing carriers and related non-carriers identified through cascade screening irrespective of their diabetes status. We focused on one variant and used a family-based design to minimise phenotypic variation associated with different genetic and environmental backgrounds. In this study carried out during more than two decades [14], we assessed the time of diabetes onset, and genetic and non-genetic factors affecting it, and explored metabolic features associated with the variant.

## Methods

The Botnia Study has been recruiting individuals with diabetes and their family members in western Finland since 1990 [19], and families with two siblings having type 2 diabetes from all of Finland during 1994–1998. Relatives and spouses without diabetes at baseline [20] as well as individuals with pathogenic MODY variants and their siblings [21] have been invited to follow-up examinations. Since 2014, the FINNMODY study has co-ordinated the study of MODY families from the Botnia Study and recruited individuals with suspected or diagnosed MODY (and family members) through: (1) advertisements directed at clinicians and patients; (2) directly contacting diabetes clinics and primary care physicians. The study doctor interviewed the potential probands.

All families consisted of at least two heterozygous carriers of the *HNF1A* p.(Gly292fs) variant and one non-carrier, who was a first-degree relative of a carrier. The 12 families with 145 carriers and 131 non-carriers (electronic supplementary material [ESM] Table 1) included three large, previously partially reported families (families B, C and D [14]), now extended by longer follow-up and new family members.

All participants gave their informed consent. The study protocol was approved by the Ethics Committees of Medicine and Paediatrics of the Helsinki University Hospital. A research nurse measured the weight, height, waist and hip circumference, heart rate, blood pressure and fat free mass. The participants filled in a questionnaire on socioeconomic and lifestyle factors, medical history and treatment. The age at diagnosis of diabetes was defined as the earliest occurrence of: (1) a diabetic glucose value at a study visit; (2) a self-reported year of the diagnosis; or (3) an ICD code for diabetes in the national registries (see ESM Methods p. 2).

Some individuals only returned the questionnaire and provided fasting blood samples, which were shipped to a central commercial laboratory (for analysis of fasting plasma glucose [FPG], HbA_1c_, alanine aminotransferase, serum creatinine, urine AER) and our research laboratory.

As a control group, we used the Prevalence, prediction and prevention of diabetes (PPP)-Botnia Study [22], conducted in the region from which most of the study participants originate (*N* = 5208; *n* = 4928 with genome-wide association study [GWAS] data).

### Metabolic characterisation

A total of 208 individuals participated in an OGTT (1.75 g/kg, maximum dose 75 g) after a 10–12 h fast at least once (109 more than once). Samples for plasma/serum glucose and insulin were drawn at 0, 30, 60, 90 and 120 min; for C-peptide, proinsulin, glucagon and NEFA, at 0 and 120 min (for a subgroup also at 30 min for C-peptide); for cholesterol, LDL- and HDL-cholesterol, triacylglycerols, creatinine, alanine aminotransferase and GAD autoantibodies, as well as blood HbA_1c_, at fasting. Young children, individuals with FPG >10 mmol/l or a diagnosis of type 1 diabetes, or those consenting only to fasting tests did not undergo an OGTT. AER was estimated from overnight urine collections. The analytic methods are described in ESM Table 2.

### Genetic testing

To determine the *HNF1A* p.(Gly292fs) (NM_00545.6:c.872dupC) variant, we sequenced exon 4 of the *HNF1A* gene by the Sanger method. Some participants had received the genetic diagnosis from the Molecular Genetics Laboratory in Exeter, UK, or the Genome Center of the University of Eastern Finland, Kuopio, Finland.

We calculated a polygenic risk score for type 2 diabetes (T2D-PRS) using GWAS data for 210 known independent type 2 diabetes risk loci according to Mahajan and colleagues [23] (details in ESM Table 3). The results were standardised to have a mean of 0 and SD of 1.

### Statistical analysis

We compared categorical variables by *χ*^2^ test with Yates’ continuity correction, and continuous variables by Mann–Whitney *U* test (MWU), reporting a standard 95% CI, *p* value and an estimator of the difference (representing the median of the difference between samples). We report nominal *p* values and comment if the statistical significance was lost after controlling for multiple comparisons (Benjamini–Hochberg procedure). Except for the analyses of age at diagnosis, all comparisons used first adult values, unless stated otherwise (ESM Table 4 shows the mean adult values during the follow-up). HOMA indices for insulin sensitivity and secretion were calculated by the HOMA2 Calculator (Oxford University 2004, www.dtu.ox.ac.uk/homacalculator [24, 25]), and composite (Matsuda) insulin sensitivity index (ISI) according to Matsuda and DeFronzo [26]. We used R (version 3.6.3) on RStudio (version 1.2.1335). See the ESM Methods (p. 2) for confirmatory and sex-specific analyses, details and a list of R packages used (p. 3).

## Results

### Onset of diabetes

**Fig. 1:**
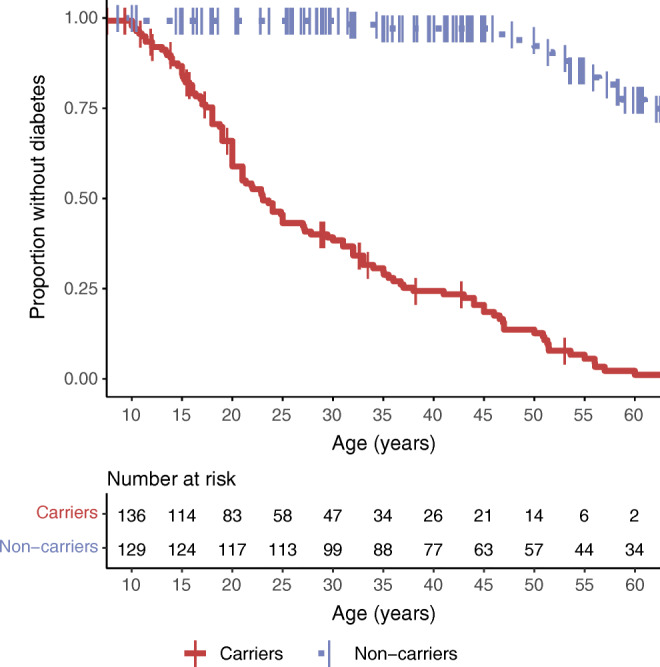
The survival analysis shows the proportion of carriers (solid red line) and non-carriers (dashed blue line) of *HNF1A* p.(Gly292fs) not diagnosed with diabetes. As compared with non-carriers, the carriers had an HR of 21 for diabetes (95% CI 12, 37; *p*<2×10^-16^)

As expected, the carriers were diagnosed with diabetes more often (83% vs 13%, *p*<2.2 × 10^−16^), and earlier (median [IQR], 21 [17–35] vs 53 [49–59]; range 7–60 vs 11–78 years; *p*=3.1 × 10^−7^), than the non-carriers. By 40 years of age, 51 of 78 (65%) carriers and one of 78 (1%) non-carriers had diabetes, and the carriers had a 51-fold increased risk of diabetes (95% CI 16, 160; *p*_Wald_=2.4 × 10^−11^, Cox proportional hazards). In a survival analysis, half of the carriers remained free of diabetes at 23 years, and one-third at 33 years (Fig. 1).

The true age at conversion to diabetes is difficult to verify retrospectively. The family members born in later decades were diagnosed younger than those born earlier, possibly because of increased awareness and systematic screening [27]. Of the 47 carriers free of diabetes (in an OGTT) at the baseline investigation at a median [IQR] age of 18 years [11–30], 21 (45%) developed diabetes during the follow-up at the age of 28 [18–47] years, the eldest at 57 years. The remaining 26 carriers had a median age of 18 [12–33] years at the last study visit, the eldest being 63 years.

We hypothesised that high BMI and poor insulin sensitivity (HOMA insulin sensitivity index [HOMA-IS], or ISI) would lower the age at diagnosis, but no significant association was seen (see ESM Results p. 4). On the contrary, among the younger carriers (aged >13 years, born after 1975) with more systematic screening, higher BMI at the first visit was associated with later age at diagnosis (Cox regression model, HR 0.86; 95% CI 0.77, 0.96; *p=*0.0066; a similar but non-significant difference was also observed using the predicted adult BMI based on the Finnish growth centiles [28]: HR 0.90; 95% CI 0.808, 1.004; *p=*0.060; *n =* 41). Rather than protection by higher BMI, more likely the defective insulin secretion resulted in lower BMI and earlier diagnosis among the carriers. This is supported by the fact that the BMI association disappeared after adjusting for fasting C-peptide (data not shown). After the diagnosis of diabetes, BMI was weakly positively associated with higher HbA_1c_ (linear regression model: 1.06 mmol/mol [0.098%] higher median HbA_1c_ for each unit of median BMI, *p=*0.00072, *n =* 107).

As diabetes was relatively prevalent also in the non-carriers, we hypothesised that genetic susceptibility to type 2 diabetes might lower the age at onset of diabetes. Indeed, the study participants had a higher T2D-PRS (median 0.10; range −2.4 to 3.6; IQR −0.52 to 0.94) than the population-based control group (median −0.015; range −3.63 to 3.55; IQR −0.70 to 0.65; between-group difference 0.20; 95% CI 0.074, 0.325; *p=*0.0019). The T2D-PRS was similar in carriers and non-carriers (*p=*0.84). T2D-PRS contributed to the onset of (any) diabetes among the participants: in a Cox proportional hazards model, 1 SD unit increase in T2D-PRS increased the risk of diabetes by 28% (HR 1.28; 95% CI 1.065, 1.535; *p*=0.00841; with the *HNF1A* carrier status as a significant covariate: HR 22.1, *p*<2 × 10^−16^). Among the carriers, for 1 SD unit increase of T2D-PRS, the predicted age at diagnosis decreased by 3.2 years in a linear model (also adjusted for the year of birth). See ESM Results (p. 5) for further analyses.

Of note, neither sex (data not shown) nor parental inheritance significantly modified the age at diagnosis (those with paternal inheritance had an insignificant 0.79-fold risk of diabetes compared with those with maternal inheritance; 95% CI 0.47, 1.30; Cox proportional hazards).

### Metabolic characterisation

Plasma glucose was higher and serum insulin and proinsulin concentrations lower in carriers than in non-carriers at fasting and during the OGTT (Table 1, ESM Table 4), compatible with insulin deficiency. The proinsulin:insulin ratio was similar in both groups (ESM Fig. 1), confirming some [14, 29] but not all [30] previous reports. The difference in insulin response was greatest at 30 min, presumably representing a defective first-phase insulin secretion among the carriers [31], and the difference in glucose levels peaked at 90 min. The difference in insulin and proinsulin response was reflected in the C-peptide concentrations at 30 min but not at 120 min. Further, a regression model (ESM Table 5) showed a diminished glucose responsiveness of insulin secretion in carriers compared with non-carriers (i.e., the non-carriers could produce high levels of serum insulin as a response to high plasma glucose). We also analysed the data restricting the analysis to the last non-diabetic visit of the carriers (*n =* 23–27) and sex- and age-matched non-carriers. The differences in glucose, insulin and C-peptide response profiles were similar but smaller than in the main analysis (only the differences in 30 and 60 min insulin were statistically significant).

**Table 1:** Characteristics of the carriers and non-carriers of *HNF1A* p.(Gly292fs)

Characteristic	Carriers			Non-carriers			Statistics			
	Result	Age at investigation	*n* (% female)	Result	Age at investigation	*n* (% female)	MWU estimator	95% CI	*p* value	LM estimate^a^
HbA_1c_ (mmol/mol)	51 [41–63]	39 [30–55]	113 (51)	36 [32–39]	41 [30–54]	100 (43)	15.2	11.2, 18.6	8.9 × 10^−21^	18
HbA_1c_ (%)	6.8 [5.9–7.9]			5.4 [5.1–5.7]						
Fasting values^b^										
Glucose (mmol/l)	7.0 [5.2–9.4]	38 [29–54]	115 (51)	5.4 [5.2–5.8]	42 [31–55]	120 (46)	1.5	0.9, 2.2	1.6 × 10^−8^	2.3
Insulin (pmol/l)	29 [17–46]	39 [29–54]	113 (51)	34 [21–50]	43 [31–55]	118 (46)	−4.4	−9.5, 0.5	0.075	−4.6
C-peptide (nmol/l)	0.30 [0.19–0.45]	38 [30–54]	99 (51)	0.36 [0.17–0.55]	41 [30–57]	97 (45)	0.0	−0.1, 0	0.24	−0.065
Insulin:C-peptide	95 [63–128]	40 [30–54]	98 (50)	117 [78–228]	41 [30–57]	96 (46)	−23	−41, −5	0.010	(−41)^c^
Proinsulin (pmol/l)	3.9 [2.8–7.3]	48 [31–62]	58 (55)	6.7 [3.6–14.8]	53 [38–65]	45 (33)	−2.4	−4.5, −0.7	0.0044	−5.9
OGTT glucose (mmol/l)										
0 min	6.2 [5.1–7.5]	36 [28–47]	72 (54)	5.4 [5.1–5.8]	42 [32–54]	107 (47)	0.7	0.3, 1.2	0.00066	1.1
30 min	10.3 [8.8–12.7]	36 [28–49]	74 (54)	8.3 [7.4–9.2]	42 [32–53]	108 (46)	2.2	1.5, 3.0	1.3 × 10^−9^	2.6
60 min	13.2 [9.8–16.5]	36 [29–49]	74 (54)	7.5 [6.3–9.8]	42 [32–53]	108 (46)	5.3	4.1, 6.5	9.6 × 10^−16^	5.6
90 min	14.9 [9.6–18.0]	40 [30–52]	59 (54)	6.2 [5.0–7.9]	44 [32–55]	75 (47)	7.70	5.8, 9.4	2.4 × 10^−13^	7.5
120 min	11.3 [8.6–16.7]	36 [28–47]	72 (54)	5.8 [5.0–7.0]	42 [32–54]	107 (47)	5.6	4.3, 7.5	5.0 × 10^−17^	6.9
OGTT insulin (pmol/l)										
0 min	27 [16–35]	36 [27–51]	70 (53)	34 [20–49]	43 [32–54]	106 (47)	−7	−13, −2	0.0057	−10
30 min	100 [51–144]	36 [27–51]	70 (53)	278 [171–437]	42 [32–54]	107 (47)	−177	−228, −133	5.7 × 10^−17^	−229
60 min	119 [79–167]	36 [28–51]	70 (53)	331 [183–447]	42 [32–54]	107 (47)	−185	−237, −138	4.0 × 10^−15^	−264
90 min	133 [89–219]	44 [30–56]	58 (52)	334 [197–484]	44 [32–55]	75 (47)	−175	−241, −116	4.6 × 10^−9^	−271
120 min	102 [60–160]	36 [27–51]	70 (53)	192 [105–318]	42 [32–54]	106 (47)	−77	−116, −45	1.7 × 10^−6^	−146
OGTT C-peptide (nmol/l)										
0 min	0.35 [0.21–0.45]	36 [29–49]	71 (54)	0.36 [0.17–0.55]	41 [30–57]	92 (48)	0.00	−0.1, 0.0	0.5	−0.052
30 min	0.75 [0.41–0.99]	36 [29–50]	69 (54)	1.22 [0.53–1.89]	41 [30–57]	93 (47)	−0.53	−0.8, −0.3	9.6 × 10^−6^	−0.55
120 min	0.96 [0.59–1.52]	36 [29–49]	71 (54)	1.30 [0.62–2.06]	41 [30–57]	92 (48)	−0.30	−0.5, 0.0	0.057	−0.38
OGTT proinsulin (pmol/l)										
0 min	3.7 [2.4–5.8]	46 [30–58]	41 (49)	6.7 [3.7–14.6]	54 [38–66]	43 (35)	−3.0	−5.1, −1.3	0.0003	−5.3
120 min	14 [10–24]	46 [30–58]	41 (49)	41 [28–74]	54 [38–66]	43 (35)	−25	−35.8, −16.9	1.2 × 10^−9^	−37
OGTT insulin:C-peptide										
30 min	152 [120–188]	36 [29–51]	67 (54)	273 [191–565]	41 [30–57]	92 (48)	−111	−156, −76	2.8 × 10^−10^	(−326)
120 min	115 [85–149]	36 [29–50]	69 (54)	135 [103–341]	41 [30–57]	91 (48)	−33	−62, −11	0.002	(−222)
Glucagon (μmol/l)										
At fasting^b^	5.5 [3.7–8.7]	46 [30–60]	48 (50)	6.4 [4.7–8.4]	54 [39–66]	39 (33)	−0.6	−2, 0.7	0.4	0.15
0 min (OGTT)	5.5 [3.7–8.0]	46 [29–57]	39 (49)	6.4 [4.7–8.4]	54 [39–66]	39 (33)	−0.9	−2.4, 0.4	0.18	−0.092
120 min (OGTT)	3.6 [2.5–5.3]	46 [29–57]	39 (49)	3.5 [2.2–5.5]	55 [40–67]	38 (32)	0.2	−0.8, 1.2	0.64	0.88
Indices of glucose metabolism										
CIR^d^	19 [9–42]	36 [27–51]	69 (54)	115 [70–173]	42 [32–54]	106 (46)	−86	−108, −68	3.3 × 10^−19^	(−115)^c^
HOMA2-B (%)	35 [19–57]	39 [29–55]	108 (52)	62 [48–78]	43 [31–55]	117 (45)	−26.6	−32.8, −20.4	6.0 × 10^−12^	−27
HOMA2-IS (%)	139 [91–232]	39 [29–55]	108 (52)	136 [90–220]	43 [31–55]	117 (45)	9.1	−13.8, 33.5	0.43	8.1
ISI^d^	9.3 [6.1–14.2]	35 [27–48]	66 (55)	6.6 [4.6–10.8]	43 [32–55]	105 (47)	2.57	1.1, 4.1	0.0012	2.8

The data represent the first adult levels (median [IQR]) of plasma glucose, serum insulin, serum C-peptide, serum proinsulin and serum glucagon at fasting, as well as during an OGTT for a subgroup. *N* (% female), total number of individuals (the proportion of women). For more results, see ESM Table 4

^a^Linear model estimate adjusted for age and sex

^b^Including the 0 min values reported for the OGTT

^c^One outlier was excluded from a linear model for CIR and nine for fasting insulin:C-peptide ratio

^d^CIR (at 30 min) in 100 pmol/l (mmol/l)^−2^, ISI in 10,000 (mg/dl)^−1^ (mU/l)^−1^; to convert ISI into 10,000 (mmol/l)^−1^ (pmol/l)^−1^, please multiply by 2.59

CIR, corrected insulin response; HOMA2-IS, HOMA2 insulin sensitivity index; LM, linear model

Although the carriers had significantly better insulin sensitivity (ISI, Table 1), a multivariate linear model showed that the difference was more strongly associated with BMI than the variant carrier status (data not shown).

### Body composition and lipidaemia

**Fig. 2:**
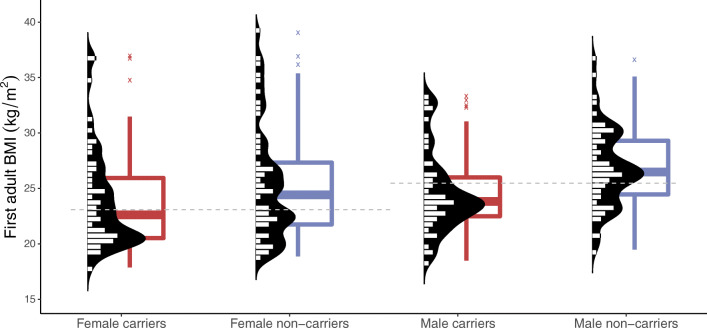
First adult BMI illustrated by a boxplot and overlapping bean plot for female and male carriers and non-carriers of the *HNF1A* p.(Gly292fs) variant. Each observation is plotted by a short horizontal line (double width symbolises two samples with the same value, etc.); the dashed grey lines represent sex-specific medians. Outliers are marked with 'X'

The adult carriers were significantly leaner than the non-carriers (Fig. 2, ESM Tables 4, 6), with a similar height but 5.0 kg lower weight (*p*=0.012) and 2.1 kg/m^2^ lower BMI (*p*=2.2 × 10^−4^). The age- and sex-adjusted difference was −4.6 kg for weight (*p*=0.0059) and −1.8 kg/m^2^ for BMI (*p*=0.0014) (linear regression model). Similarly, the carriers were leaner than age- and sex-matched individuals from the population-based PPP-Botnia Study (−1.67 kg/m^2^ with *p=*0.0030, *n* = 107, matched for the closest possible age, median age 38.0 vs 38.0).

The differences in body composition between carriers and non-carriers were significant in men but the results in women were in the same direction (weight: −6.9 kg [95% CI −11, −2.2], *p=*0.0047 vs −2.5 kg [−6.0, 2.7], *p*=0.41; BMI: −2.5 kg/m^2^ [−3.7, −1.2], *p*=2.4 × 10^−4^ vs −1.3 kg/m^2^ [−2.7, 0. 25], *p*=0.10). The difference was also attenuated regarding the latest ‘non-diabetic’ BMI (last measurement before diabetes or last ever in case of no diabetes): the median BMI of the carriers (*n =* 51) vs age- and sex-matched non-carriers (*n =* 51) was 21.2 vs 22.1 kg/m^2^ (MWU estimate −1.4 kg/m^2^, *p*=0.11, matched for the closest possible age, median age 18 vs 21).

Of note, the weight difference was equally reflected in fat and lean body mass. In linear models adjusted for sex, age and height, the carriers had 3.0 kg lower body fat mass (*p*=0.0029), and 1.6 kg lower lean body mass (*p*=0.049, NS). The difference in fat mass was significant only in men (ESM Table 6).

**Fig. 3:**
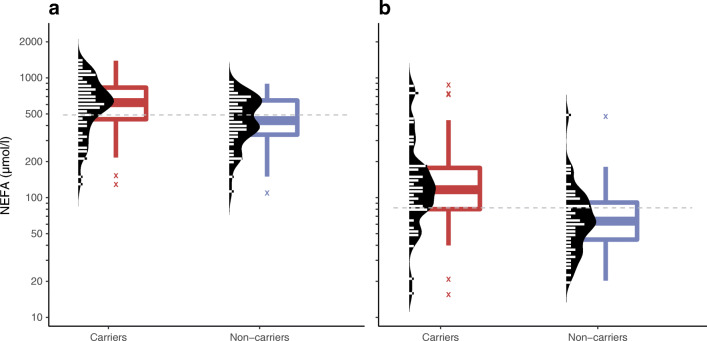
First measurements in adult age of NEFA at fasting (**a**) and at 120 min during an OGTT (**b**) in carriers and non-carriers of the *HNF1A* p.(Gly292fs) variant. Each observation is plotted by a short horizontal line (double width symbolises two samples with the same value, etc.); the dashed grey lines represent medians. Also, a sex- and age-adjusted log-transformed linear regression model implied lower NEFA levels among the carriers compared with the non-carriers both at fasting (*p*=0.00050) and at 120 min (*p*=2.2×10^-7^) after excluding the outliers marked with ‘X’. One non-carrier outlier with fasting NEFA >2000 μmol/l was excluded from the figure

We hypothesised that insulin deficiency associated with the *HNF1A* defect would lead to increased lipolysis. Accordingly, the carriers had higher levels of NEFA than the non-carriers both at fasting (median [IQR] 621 [452–829] vs 441 [340–648] μmol/l, MWU: *p*=0.0039, *n =* 64 vs 50) and at 120 min (117 [80–177] vs 64 [45–91] μmol/l, MWU: *p*=3.1 × 10^−5^, *n =* 46 vs 47; Fig. 3).

The groups had similar lipid levels except for HDL-cholesterol concentration, which was higher in male carriers than non-carriers (median [IQR] 1.41 [1.14–1.54] vs 1.20 [1.05–1.38], *p*=0.0051) (ESM Tables 4, 6). Apo-CIII levels were similar (ESM Table 6).

### Glucagon

**Fig. 4:**
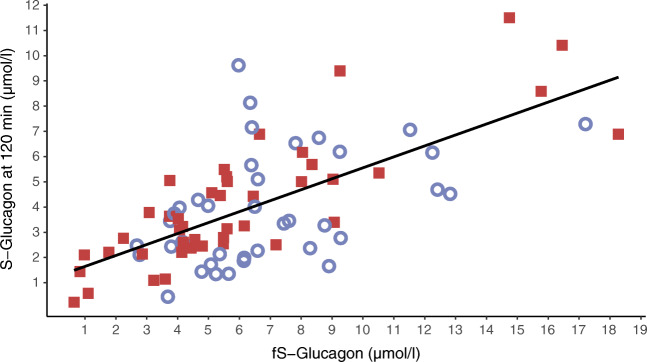
Fasting glucagon plotted against 120 min glucagon in 43 carriers (red square boxes) and 38 non-carriers (blue circles) of the *HNF1A* p.(Gly292fs) variant, with a regression line for the combined group (*p*=4.5×10^-12^, *r*^2^ 0.45). fS-glucagon, fasting serum glucagon; S-glucagon, serum glucagon

Impaired suppression of glucagon secretion by glucose has been reported in HNF1A-MODY [32]. In our families, glucagon levels were similar in carriers and non-carriers, both at fasting and at 120 min (Fig. 4, Table 1), questioning a direct effect of the variant on alpha cells. Unfortunately, only one of the carriers with glucagon data was free of diabetes at sampling.

### Urine threshold for glucose

The urine threshold for glucose did not clearly differ between the carriers and the non-carriers (data in the ESM Results p. 6).

### Is there a cut-off for insulin or C-peptide to exclude MODY?

The highest insulin levels in our carriers were slightly below 400 pmol/l after age 35, and up to 567 pmol/l during normoglycaemia and 475 pmol/l during dysglycaemia in younger carriers. Fasting C-peptide was below 1.0 nmol/l in all carriers irrespective of glucose tolerance and age, but post-challenge concentrations reached 3.02 nmol/l in young carriers and 2.4 nmol/l in >35-year-old carriers with dysglycaemia. On the other hand, the all-time lowest fasting C-peptide levels typically exceeded 0.2 nmol/l (79% of the measurements) but had been <0.1 nmol/l in 11/125 carriers.

### The participation rate and the age at entering the study

The three large families (B, C and D) constituted the major study population, with detailed data on multigenerational family structure. To ensure that participation bias did not affect the results, we evaluated the participation rate, i.e., the proportion of the participants of all potential participants (the actual participants and their non-participating siblings and the carriers’ offspring), in them. The overall participation rate was 74% in the main and 85% in the siblings-only analysis. The corresponding figures were 84% and 94% among those born in 1975–2000. Most siblings of those born since the 1960s participated (ESM Fig. 2a). The carrier:non-carrier ratio was 53%:47%, close to the expected 50%:50% (*p*=0.15).

The median age at the first visit depended on the decade of birth (ESM Fig. 2b), and has gradually fallen from around 70 years among those born in the 1920s, to under 20 years in those born in the 1980s or later. Thus, the majority of those born before the 1980s had been diagnosed with diabetes already at the first visit, whereas most carriers born later had been free of diabetes at their first visit.

## Discussion

In this family-based study comparing relatives with and without the heterozygous *HNF1A* variant p.(Gly292fs), we show that although half of the variant carriers progress to overt diabetes by the age of 23 years, 13% are free of diabetes at the age of 50. In addition, T2D-PRS modified the age at onset of diabetes. The relative insulin deficiency is characterised by hyperglycaemia, lipolysis and markedly lower adult BMI. Studying individuals based on genotype rather than phenotype bypasses some problems with ascertainment bias and offers a more realistic view of the manifestations of the gene variant.

Presentation at young age has been one of the cornerstones of the diagnosis of MODY. The diagnosis age of the probands in the original studies [3, 4, 33] guided the early clinical criteria to include age at onset before 25 years in at least 1–2 family members [34]. In a larger subsequent series, 70% of the participants with HNF1A-MODY were diagnosed with diabetes by the age of 25 and 85% by 35 years [35]. However, these figures only pertain to individuals who developed diabetes, and mostly were clinically suspected to have MODY, biasing towards a more severe phenotype. In contrast, we invited all relatives regardless of their glycaemic status to participate in the study, combining retrospective, cross-sectional and prospective follow-up data. The penetrance was lower than in the clinical series: 57% of carriers had converted to diabetes before the age of 25 and 70% by 35 years. Of note, 13% were still free of diabetes at the age of 50.

While we observed no effect of BMI, insulin sensitivity, parental carrier status or sex on age at diagnosis, the T2D-PRS, based on 210 known risk loci for type 2 diabetes, advanced the onset of diabetes moderately. In a Cox model, +1 SD of T2D-PRS increased the risk of diabetes by 28%, whereas in a linear regression model +1 SD of T2D-PRS was associated with a 3 year earlier diagnosis of *HNF1A*-diabetes. Previously, using 17-SNP T2D-PRS, Lango Allen and colleagues reported that each additional SNP was associated with a 0.35 year earlier diagnosis in HNF1A-MODY [36]. Our data support adding HNF1A-MODY to the diseases in which the polygenic risk score modulates the manifestation of monogenic variants [37].

Identification of carriers before they developed diabetes enabled us to explore early phenotypes and to compare them with genetically and environmentally matched control participants, i.e., related non-carriers. The high participation rate and similar T2D-PRS distribution in the groups minimised the ascertainment bias. Untargeted population-wide sequencing might further reduce it [38], considering that a suspicion of MODY had motivated the genetic testing of the first members. Also, the higher T2D-PRS than in the background population might have aided in recognising the high familial prevalence of diabetes. However, as MODY often presents with slowly progressing asymptomatic hyperglycaemia, diagnostic measures in a population setting (like clinical diagnosis, HbA_1c_ or FPG) are not sensitive enough to define the time of conversion [27]. Active population screening by an OGTT would be crucial to recognise those with undiagnosed diabetes. For example, among a middle-aged population, screening revealed undiagnosed diabetes in 6.4% of women and 11.6% of men in Finland [39], and in 5.2% of women and 10.4% of men in Spain [40]. We also previously reported that the carriers born after 1975 were diagnosed with diabetes earlier than former generations, presumably due to increased awareness and screening [27]. An additional covariate of birth year also increased the statistical power of the regression models on T2D-PRS.

We could not identify clinical factors that predicted the age of conversion to diabetes. Maternal inheritance and parental age at onset of diabetes have previously been linked to the offspring’s age at diagnosis [36, 41, 42], but our study found no support for this. It has been suggested that the parent of origin plays no role as long as the offspring is not unexposed to maternal hyperglycaemia during pregnancy [41]. Therefore, maternal inheritance might only contribute to the onset age of the offspring indirectly through gestational hyperglycaemia. We also found no support for the hypothesis of insulin resistance or high BMI lowering the age at onset, agreeing with a previous report [36]. In contrast, the observed association between lower BMI and earlier diabetes among young carriers who have undergone active screening for diabetes could reflect impaired beta cell function and decreased anabolic effects of insulin, which is reinforced by the disappearance of the association by adjusting for C-peptide.

Individuals with HNF1A-MODY are leaner than individuals with type 2 diabetes [9, 12, 14, 43–46]. Whether the pathogenic *HNF1A* variants are associated with body mass in general has largely been ignored, although variant carriers have been leaner than individuals with type 1 diabetes and control individuals [14, 21, 43, 47]. Therefore, we also explored association with body composition. Variant carriers were ~5 kg (~2 kg/m^2^) leaner than their relatives, whereas adult height was similar. The difference in BMI was similar when age- and sex-matched control participants from the PPP-Botnia Study were used for the comparison (−1.67 kg/m^2^), which speaks against a collider bias.

Moreover, the differences in fat mass (3.0 kg) and lean body mass (2.6 kg) were of a similar magnitude between the carriers and non-carriers in this study, but only the fat mass difference reached statistical significance. As BMI was a stronger predictor of insulin sensitivity (ISI) than the carrier status (data not shown), *HNF1A* seems to indirectly affect insulin sensitivity through lowering BMI. Indeed, a better insulin sensitivity has been shown in carriers, whose mean BMI was lower than that of their comparators [14], and vice versa [15], while the difference in insulin sensitivity disappeared when matched for BMI [16]. Because the difference in body weight was attenuated after excluding measurements after the diagnosis of diabetes, the difference might result from progressive insulin deficiency and anabolic effect of insulin [48] or loss of energy due to glucosuria (or both).

HNF1A-MODY has been associated with a lower renal threshold for glucose and glucosuria [3, 29, 49, 50], which could result from insulin deficiency or be a direct effect of *HNF1A* through reduced renal expression of *SLC5A2* (also known as *SGLT2* for sodium–glucose cotransporter 2) [50, 51], a key transporter involved in tubular glucose reabsorption. In vitro studies indicate that the postprandial insulin surge enhances glucose reabsorption in kidneys [52]. Thus, postprandial insulin deficiency might promote glucosuria, but human data are lacking. In a previous study, all individuals with a pathogenic *HNF1A* variant and a peak plasma glucose >8.4 mmol/l demonstrated post-OGTT glucosuria [29], but our study did not replicate this finding. A combination of a graded intravenous glucose infusion with an insulin infusion, to yield matched glucose and insulin concentrations in carriers and non-carriers, might provide a definitive answer.

Consistent with insulin deficiency and less inhibition of lipolysis [53], the carriers had significantly higher NEFA levels than non-carriers both at fasting and after an OGTT. Increased lipolytic activity might contribute to the differences in body weight and fat mass. On the other hand, free circulating NEFA could in turn worsen beta cell function [54, 55].

As hyperglucagonaemia has been reported in individuals with type 1 and 2 diabetes as well as in HNF1A-MODY [32, 56, 57], we evaluated a possible relationship between lipolysis and excess glucagon, despite only vague previous data [58]. Despite the differences in glucose and insulin responses, the glucagon level was similar in the carriers and non-carriers, suggesting relative hyperglucagonaemia in the carriers (hyperglycaemia should acutely suppress glucagon secretion). Unfortunately, the cross-sectional comparison of mostly carriers with diabetes and non-carriers without diabetes precluded a reliable comparison. At present, we can only speculate that while hyperglycaemia is not a potent enough suppressor of glucagon secretion, the degree of insulin secretion was sufficient to inhibit excess glucagon secretion. Notably, a recent study on human islets with an *HNF1A* defect observed an abrogated rather than increased glucagon response from alpha cells [59].

*HNF1A* defects lead to insulin deficiency, and expectedly the carriers had lower serum insulin concentrations than the non-carriers. Confirming results from previous studies and suggestive of an impaired first-phase insulin secretion, the difference in insulin levels was most clear during an OGTT and peaked at 30 min [14, 15, 21]. Interestingly, both at fasting and during OGTT, the difference in insulin levels outweighed that in proinsulin or C-peptide levels. The higher insulin:C-peptide ratio in the carriers might be attributed to a difference in hepatic insulin clearance. Perhaps the carriers, who are leaner and thus presumably less insulin-resistant than non-carriers, can extract proportionally more insulin, or, alternatively, the *HNF1A* defect directly affects hepatic function [60].

### Conclusions

In this study based on ascertainment of participants by genotype rather than clinical presentation, one-third of the heterozygous carriers of the *HNF1A* p.(Gly292fs) variant were free of diabetes at the age of 33, and 13% at the age of 50 years. The polygenic risk for type 2 diabetes lowered the age at onset of carriers. We could not identify clinical factors affecting the age at conversion, but could exclude major effects of sex, BMI, insulin sensitivity and parental carrier status. Between the carriers and non-carriers, the fasting and OGTT measurements of serum insulin were more discriminative than those of serum C-peptide. The carriers were leaner than non-carriers and had higher lipolytic activity.

## Supplementary information

ESM (PDF 547 kb)

## Data Availability

Considering issues of patient confidentiality and restrictions in IRB permissions, original data can only be available through specific request and material transfer agreement following the EU regulations.

## Abbreviations

FPG: Fasting plasma glucose
GWAS: Genome-wide association study
ISI: Insulin sensitivity index
MWU: Mann–Whitney *U* test
PPP-Botnia Study: Prevalence, prediction and prevention of diabetes-Botnia Study
T2D-PRS: Polygenic risk score for type 2 diabetes
