# Supplementary material for: A multigenerational study on phenotypic consequences of the most common causal variant of HNF1A-MODY
Source: Diabetologia. 2021 Dec 24;65(4):632–43. doi: 10.1007/s00125-021-05631-z (PMC8894160; doi:10.1007/s00125-021-05631-z)
Supplement: Supplementary file 1 — (PDF 547 kb) [file 125_2021_5631_MOESM1_ESM.pdf]

ELECTRONIC SUPPLEMENTARY MATERIAL (ESM)

A multigenerational study on phenotypic consequences of the most common causal variant of HNF1A-MODY

ESM Methods

Research Design and Methods p. 2

Statistical analysis p. 2

The statistical libraries used in R p. 3

ESM Results

Onset of diabetes p. 4

The T2D-PRS and the onset of diabetes p. 5

Urine threshold for glucose p. 6

ESM Table 1 p. 7

ESM Table 2 p. 8

ESM Table 3 p. 10

ESM Table 4 p. 18

ESM Table 5 p. 21

ESM Table 6 p. 22

ESM Fig. 1 p. 24

ESM Fig. 2 p. 25

ESM Fig. 3 p. 26

ESM References p. 27

ESM Methods

Research Design and Methods

The onset of diabetes was defined as the earliest occurrence of i) a diabetic value at a study visit (fasting plasma glucose, FPG ≥ 7.0 mmol/l or 120-minute glucose during an oral glucose tolerance test (OGTT) > 11 mmol/l or HbA_{1c} ≥ 48 mmol/mol (6.5%); ii) a self-reported year of the diagnosis, or iii) an ICD code for diabetes in the national registries (the Care Registers for Health Care, HILMO, Finnish institute for health and welfare) from birth until five years after the last study visit (ICD9: 250*; ICD10: E10.*-E14.*, * representing any number, or H28.0, H36.0, N08.3).

Statistical analysis

We controlled the statistically significant results by Welch's *t*-test and sex-and-age-adjusted linear models (commented only in case the significance changed) using both first and mean adult values (after log-transformation, if necessary). Confirmatory *t*-testing for corrected insulin response (CIR), occasionally with negative values, was performed without transformation. All sex-specific analyses on the statistically significant observations were at least in the same direction in both sexes (expect for fasting proinsulin, which showed a negative and significant male estimate, whereas the female estimate was marginally positive but insignificant). Proinsulin values below the lowest standard of 1.775 pmol/l were adjusted at $\frac{1.775}{2}$ pmol/l. For composite (Matsuda) insulin sensitivity index (ISI), we substituted a 90-min measurement (if unavailable) with a mean of 60 and 120 minutes.The computer model-derived homeostatic model assessment (HOMA) indices (see methods) for insulin sensitivity (HOMA2 IS, an inverse number of HOMA2-IR) and insulin secretion (HOMA2β) applied a conversion factor of 6.945 for insulin (from mU/l to pmol/l) provided by the manufacturer, although similar results were obtained using an alternative conversion factor of 6.00.

The main and clearly significant results were significant also in the three large families alone. Some more subtle results required a larger sample size to reach the statistical significance level. On the other hand, in smaller families alone, the results resembled those seen in the large families (B-D), but possibly because of the low sample size, the analyses did not as often reach the significance levels.

The statistical libraries used in R:

R Core Team (2020). R: A language and environment for statistical computing. R Foundation for Statistical Computing, Vienna, Austria. URL https://www.R-project.org/.

Hadley Wickham, Romain François, Lionel Henry and Kirill Müller (2020). dplyr: A Grammar of Data Manipulation. R package version 0.8.5. https://CRAN.R-project.org/package=dplyr

H. Wickham. ggplot2: Elegant Graphics for Data Analysis. Springer-Verlag New York, 2016.

Hadley Wickham (2019). stringr: Simple, Consistent Wrappers for Common String Operations. R package version 1.4.0. https://CRAN.R-project.org/package=stringr

Alboukadel Kassambara, Marcin Kosinski and Przemyslaw Biecek (2019). survminer: Drawing Survival Curves using 'ggplot2'. R package version 0.4.6. https://CRAN.R-project.org/package=survminer

Terry M. Therneau (2020). coxme: Mixed Effects Cox Models. R package version 2.2-16. https://CRAN.R-project.org/package=coxme

Douglas Bates, Martin Maechler, Ben Bolker, Steve Walker (2015). Fitting Linear Mixed-Effects Models Using lme4. Journal of Statistical Software, 67(1), 1-48. doi:10.18637/jss.v067.i01.

Hadley Wickham (2007). Reshaping Data with the reshape Package. Journal of Statistical Software, 21(12), 1-20. URL http://www.jstatsoft.org/v21/i12/.

Bendix Carstensen, Martyn Plummer, Esa Laara, Michael Hills (2019). Epi: A Package for Statistical Analysis in Epidemiology. R package version 2.40. URL https://CRAN.R-project.org/package=Epi

ESM Results

Onset of diabetes

The association of the carriers' age at diagnosis of diabetes with BMI and indices for insulin sensitivity by Cox regression:

a) Using only adult measurements
   a. Composite index (Matsuda) for insulin sensitivity (57 diagnosed with diabetes, HR 1.001, CI 0.998; 1.003, p = 0.6)
   b. HOMA2 insulin sensitivity index (99 diagnosed with diabetes, HR 1.000, CI 0.999; 1.002, p = 1)
   c. BMI (96 diagnosed with diabetes, HR 1.02, CI 0.967; 1.071, p = 0.5)
b) Including also measurements before 18 years of age
   a. Composite index (Matsuda) for insulin sensitivity (69 diagnosed with diabetes, HR 0.999, CI 0.997; 1.002, p = 0.6)
   b. HOMA2 insulin sensitivity index (113 diagnosed with diabetes, HR 0.999, CI 0.998; 1.001, p = 0.3)
   c. BMI (109 diagnosed with diabetes, HR 0.959, CI 0.910; 1.01, p = 0.1)
   d. BMI (adjusted to adult equivalent (as described by [28]) for those under 18 years old): no relevant changes vs. the results above

No significant differences were seen even if we replaced the measurements at the time of the diagnosis (or as close as possible) with the first or last measured value during the follow-up, with or without limiting to the values before the diagnosis of diabetes. Also, when further performing mixed effects Cox models, none of the models yielded an Akaike information criterion to support that BMI or the indices above would affect the age at onset.

The T2D-PRS and the onset of diabetes

To increase the power of the Cox proportional hazards models, we also included the year of birth as a covariate, because the younger participants have undergone more active screening for diabetes than the older ones. Inclusion of the birth year as a covariate in a Cox proportional hazards model increased the statistical power of the analyses:

1. In a model with both carriers and non-carriers, T2D-PRS had β = 0.352 (HR 1.42) with p = 0.00021, *HNF1A* carrier status β = 3.07 (HR 21.6) with $p < 2\times10^{-16}$, and year of birth β = 0.031 (HR 1.03) with $p = 9.4\times10^{-9}$
2. In a model with only carriers, T2D-PRS had β = 0.318 (HR 1.38) with p = 0.0019, and the year of birth β = 0.028 (HR 1.03) with $p = 6.7\times10^{-7}$

Similarly, a linear model without any covariate but only to include the carriers born since 1930, the result was of the same magnitude (for 1 SD unit increase of T2D-PRS, predicted age at diagnosis decreased by 3.0 years, p = 0.0254, r^2 = 0.037).

Among the carriers, those diagnosed with diabetes before the age of 20 years had a significantly higher T2D-PRS than those remaining free of diabetes at 20 years (mean 0.54 vs 0.070; MWU estimator -0.50; CI -0.89, -0.13; p = 0.0091). The carriers belonging to the highest tertile of T2R-PRS had an earlier onset of diabetes than the carriers in the lowest tertile (mean 24 yrs vs 31 yrs; MWU estimator -6.1 yrs, CI -13.0, -1.1, p=0.016).

Urine threshold for glucose

HNF1A variant carriers have been reported to have lower renal glucose threshold than related non-carriers [ESM1]. We compared urine glucose excretion (dipstick data detecting U-glucose $\geq$5.5 mmol/l) during the OGTT in carriers and non-carriers without measurable fasting urine glucose. We found no difference in the relationship between the urine glucose and P-glucose AUC (ESM Fig. 3) or peak P-glucose (data not shown) between groups. In three carriers, P-glucose peaked relatively high (> 8.5 mmol/l) during the OGTT without positive U-glucose after the OGTT. Also, negative fasting U-glucose result was observed despite FPG as high as 13.4 mmol/l in one carrier, and between 7.4 and 9.9 mmol/l in 12 carriers.

ESM Table 1. The participating families

Family	Carriers (F/M)	Non-carriers (F/M)
C	49 (26/23)	47 (21/26)
D	44 (23/21)	45 (18/27)
B	16 (9/7)	17 (9/8)
E (new)	5 (4/1)	3 (2/1)
F (new)	3 (2/1)	1 (1/0)
G (new)	2 (0/2)	2 (0/2)
H (new)	4 (2/2)	2 (2/0)
I (new)	3 (2/1)	2 (1/1)
J (new)	2 (1/1)	4 (1/3)
K (new)	4 (2/2)	1 (1/0)
L (new)	5 (2/3)	2 (0/2)
M (new)	8 (6/2)	5 (3/2)
	**145 (79/66)**	**131 (59/72)**

ESM Table 1 shows the number of carriers and non-carriers of the *HNF1A* p.(Gly292fs) variant in the included 12 families. The three largest families have been previously partially published (families B, C, and D, coded as in [14]). The numbers include both paediatric and adult participants. F; female, M; male.

ESM Table 2. The analytic methods

Substance/measure	Assay/method	Note
P- Glucose	Glucose oxidase method (Beckman Glucose Analyzer, Beckman Instruments, Fullerton, CA, USA), and since 2010, Hemocue Glucose System, HemoCue, Ängelholm, Sweden)	Blood glucose measured by hexokinase method (Boehringer Mannheim, Mannheim, Germany) in the early years of study [14] was converted to plasma glucose by a factor of 1.13.
S- Insulin	Radioimmunoassay (Linco, Pharmacia, Uppsala, Sweden), enzyme immunoassay (DAKO, Cambridgeshire, U.K.), fluoroimmunometric assay (AutoDelfia, Perkin Elmer Finland, Turku), electrochemiluminometric immunoassay (Cobas e411, Roche, Mannheim, Germany)	The linear conversion factors were 0.801 from DAKO to AutoDelfia, and 1.142 from AutoDelfia to Cobas. The conversion from Linco to DAKO was based on a linear piecewise formula. The conversion factor from mU/l to pmol/l was 6.945.
S- C-peptide	Radioimmunoassay (Linco, Pharmacia, Uppsala, Sweden), fluoroimmunometric assay (AutoDelfia, Perkin Elmer Finland, Turku), electrochemiluminometric immunoassay (Cobas e411, Roche, Mannheim, Germany)	A non-linear conversion from Linco to AutoDelfia was based on a formula $0.802 \times 2.885^{\log(C-peptide(Linco))}$, whereas AutoDelfia to Cobas required no conversion.
S- Glucagon	Enzyme immunoassay (Mercodia, Uppsala, Sweden)	
S- Proinsulin	Enzyme immunoassay (Mercodia, Uppsala, Sweden)	
S- NEFA	Enzymatic colorimetric method (Wako Chemicals, Neuss, Germany)	
S- Apo-C3	Immunoturbidimetric method (Kamiya Biomedical, Seattle, USA)	
S -Cholesterol, S- HDL cholesterol, S- Triglycerides	Enzymatic method (CobasMira analyser; Hoffman LaRoche, Basel, Switzerland)	LDL cholesterol calculated by the Friedewald formula
S -Cholesterol, S- HDL cholesterol, S- LDL-cholesterol, S-Triglycerides	Enzymatic method (Konelab 60i analyser and Indiko plus analyser; Thermo Electron, Vantaa, Finland)	

U- Glucose	Semiquantitatively method with reagent sticks (Uristix, Siemens, or Diastix, Bayer).	
Fat free mass	Bioelectrical impedance technique with Tanita Body Composition Analyzer BF-350, Tanita Europe GmbH, and until 2004, an infrared spectroscopy technique with Futrex 5000, Futrex, Gaithersburg, Md. on the outer layer of the biceps	

As the results from the four insulin assays as well as the three C-peptide assays correlated strongly with each other (with r^2 at 96% or more), respectively, the measurements for insulin and C-peptide were transformed to cohere with those obtained from the electrochemiluminometric immunoassay. We excluded data for samples from earlier (1990s) visits analysed by less specific methods without reliable conversion factors. P, plasma; S, serum; U, urine.

ESM Table 3. The SNPs, their effect allele and effect size (β) used for the construction of the polygenic risk score for type 2 diabetes (T2D-PRS)

Chromosome	Position (build 37)	Effect allele	Other allele	β	*p* value
1	40035928	T	G	0.085	1.30×10^{-26}
1	51440093	T	C	-0.069	5.90×10^{-10}
1	62579891	T	G	-0.064	1.20×10^{-08}
1	120437884	A	G	-0.082	5.60×10^{-16}
1	120505532	A	G	0.072	6.40×10^{-14}
1	177913519	T	C	-0.051	1.40×10^{-10}
1	206595069	T	C	0.036	2.70×10^{-08}
1	214159256	T	C	-0.068	5.60×10^{-26}
1	214272242	A	C	-0.065	1.60×10^{-03}
1	219748818	C	G	0.057	3.70×10^{-16}
1	235688043	A	G	0.039	4.00×10^{-09}
2	417167	T	C	0.11	3.30×10^{-11}
2	653575	T	C	-0.056	5.90×10^{-11}
2	27730940	T	C	-0.067	1.30×10^{-24}
2	43215855	T	C	-0.026	5.40×10^{-05}
2	43436754	A	G	0.036	1.90×10^{-08}
2	43687879	T	C	-0.12	5.30×10^{-30}
2	58981325	A	G	-0.036	4.40×10^{-08}
2	59307725	A	G	-0.037	1.70×10^{-08}
2	60584819	A	G	0.058	1.20×10^{-19}
2	65284623	T	C	0.044	2.60×10^{-11}
2	103054449	A	G	-0.012	1.10×10^{-01}
2	121383184	A	G	0.079	2.10×10^{-05}
2	147855299	T	C	-0.052	9.40×10^{-09}
2	165513091	T	C	0.06	1.60×10^{-20}
2	165573194	T	C	0.055	3.80×10^{-04}

2	227092802	A	G	-0.094	1.50×10^{-45}
3	12329783	T	C	-0.11	2.80×10^{-27}
3	12489342	T	G	-0.031	1.10×10^{-06}
3	23454790	A	G	0.071	1.10×10^{-19}
3	23635623	A	G	-0.12	7.40×10^{-11}
3	46925539	T	C	-0.038	1.50×10^{-08}
3	53125585	T	C	-0.035	3.90×10^{-08}
3	54823598	A	G	-0.07	1.80×10^{-07}
3	64002897	T	C	0.048	2.70×10^{-08}
3	123065778	A	G	0.089	1.30×10^{-31}
3	129331906	A	C	0.048	1.30×10^{-06}
3	152382636	A	G	0.03	1.60×10^{-05}
3	152535365	T	C	0.09	3.10×10^{-08}
3	168219437	T	C	-0.051	2.50×10^{-08}
3	170724883	T	C	0.065	3.20×10^{-20}
3	183708439	T	C	0.032	3.20×10^{-07}
3	185508591	T	C	-0.11	5.10×10^{-58}
3	185548683	A	G	0.039	3.30×10^{-09}
3	186665645	T	C	0.055	1.40×10^{-17}
3	186668108	A	G	-0.035	6.10×10^{-03}
3	187740523	T	C	-0.06	4.70×10^{-20}
4	727695	A	C	-0.11	6.60×10^{-12}
4	6289986	T	G	-0.086	1.20×10^{-39}
4	17812615	A	C	-0.037	2.60×10^{-07}
4	45182527	A	G	-0.044	6.80×10^{-12}
4	52784678	A	G	-0.035	8.40×10^{-07}
4	89741269	A	G	0.035	6.60×10^{-08}
4	95026434	A	T	-0.038	2.50×10^{-09}
4	104140848	A	C	-0.041	2.90×10^{-10}
4	137094047	A	C	-0.035	6.30×10^{-08}

4	153520475	T	C	-0.055	3.20×10^{-14}
4	157720253	T	C	0.036	1.30×10^{-07}
4	185714289	T	G	-0.069	1.40×10^{-13}
5	44446133	T	C	-0.073	2.00×10^{-04}
5	53271420	A	G	0.051	2.10×10^{-13}
5	55806751	A	G	-0.073	4.40×10^{-23}
5	55841462	A	G	-0.022	6.20×10^{-04}
5	55861601	A	G	0.067	6.10×10^{-17}
5	56196604	A	G	-0.038	1.70×10^{-07}
5	67714246	A	G	-0.035	1.00×10^{-07}
5	75003678	T	C	0.053	3.30×10^{-16}
5	76424949	A	G	-0.059	1.40×10^{-17}
5	78416416	T	C	-0.044	4.60×10^{-11}
5	86610989	A	C	-0.037	7.30×10^{-07}
5	102338811	A	G	-0.16	1.20×10^{-28}
5	133399909	T	C	-0.027	1.30×10^{-04}
6	7214499	T	C	-0.04	1.10×10^{-06}
6	20679709	A	G	-0.14	3.00×10^{-87}
6	32583357	A	T	-0.078	5.00×10^{-20}
6	34207990	T	C	0.076	1.30×10^{-06}
6	43757896	A	C	0.041	6.00×10^{-10}
6	43811762	T	C	0.051	6.90×10^{-13}
6	50816887	T	G	0.059	1.20×10^{-12}
6	107431688	A	G	-0.039	1.00×10^{-08}
6	127412728	A	G	-0.058	1.10×10^{-14}
6	137300960	A	G	0.041	7.60×10^{-09}
6	139831180	A	G	-0.033	3.30×10^{-07}
6	160769423	A	G	-0.037	4.00×10^{-09}
7	14898282	T	C	0.069	6.90×10^{-17}
7	15065003	T	C	-0.066	3.20×10^{-25}

7	15191273	T	C	0.019	6.60×10^{-03}
7	28198677	T	C	-0.092	4.20×10^{-48}
7	30726777	A	T	0.044	7.70×10^{-09}
7	102471780	T	C	0.046	3.20×10^{-08}
7	103417999	T	C	-0.035	7.80×10^{-08}
7	150542515	T	G	0.039	9.50×10^{-09}
8	19830921	T	C	-0.07	8.70×10^{-13}
8	41509083	T	C	0.1	1.00×10^{-09}
8	41509259	A	G	0.063	3.80×10^{-22}
8	110122139	T	C	-0.035	3.70×10^{-08}
8	118185025	A	G	-0.11	6.30×10^{-55}
8	129568078	T	C	-0.043	1.90×10^{-09}
8	145509128	T	C	0.05	9.80×10^{-14}
8	145904423	A	C	-0.036	4.80×10^{-08}
9	4291928	A	C	-0.051	1.60×10^{-14}
9	20714014	T	C	0.041	4.80×10^{-06}
9	22133984	T	C	-0.021	1.10×10^{-03}
9	22134094	T	C	0.16	8.20×10^{-79}
9	22141269	T	C	-0.014	4.10×10^{-02}
9	22301092	A	T	-0.077	2.00×10^{-05}
9	28414339	A	G	-0.041	3.00×10^{-09}
9	81365721	A	G	-0.033	2.70×10^{-07}
9	81905590	A	G	0.1	2.90×10^{-14}
9	84308948	A	G	-0.066	8.50×10^{-24}
9	139245289	A	G	-0.068	2.40×10^{-18}
10	12307894	T	C	0.09	3.70×10^{-32}
10	71466578	T	G	-0.052	6.30×10^{-14}
10	80943841	A	G	-0.071	5.90×10^{-28}
10	81091563	A	C	0.029	1.50×10^{-04}
10	89768584	A	G	0.044	8.50×10^{-07}

10	93930279	T	C	-0.049	2.50×10^{-14}
10	94465559	T	C	-0.11	2.70×10^{-62}
10	114686805	T	C	-0.0024	7.10×10^{-01}
10	114754071	T	C	-0.31	5.8×10^{-447}
10	114797893	A	G	-0.089	6.70×10^{-25}
10	114871594	A	G	0.034	2.50×10^{-06}
10	124192430	T	C	0.046	2.20×10^{-13}
11	2195981	T	C	0.069	1.90×10^{-24}
11	2390172	T	C	0.03	1.10×10^{-04}
11	2579163	A	G	0.066	1.40×10^{-05}
11	2673681	T	C	0.049	9.70×10^{-07}
11	2850782	T	C	-0.029	1.50×10^{-04}
11	2857194	A	C	-0.093	3.60×10^{-44}
11	2858440	A	G	-0.19	1.60×10^{-29}
11	17408630	T	C	-0.07	2.00×10^{-26}
11	32956492	T	C	-0.11	3.90×10^{-11}
11	34643721	T	C	0.041	4.20×10^{-06}
11	34968523	A	G	0.039	9.30×10^{-08}
11	43877934	A	C	0.05	8.50×10^{-13}
11	47529947	A	C	0.037	6.40×10^{-09}
11	65294799	T	C	0.061	1.40×10^{-14}
11	72433098	A	C	0.1	2.70×10^{-32}
11	92708710	C	G	-0.099	1.50×10^{-43}
11	93013387	T	G	-0.044	8.80×10^{-10}
11	128234144	A	G	0.045	2.00×10^{-10}
12	4020527	T	C	-0.035	9.30×10^{-06}
12	4297831	A	C	0.046	2.70×10^{-08}
12	4374373	A	G	-0.056	7.40×10^{-13}
12	12871099	T	G	-0.044	3.50×10^{-08}
12	26472562	A	G	0.047	1.60×10^{-10}

12	27965150	T	C	-0.074	2.50×10^{-20}
12	66212318	T	C	0.11	6.60×10^{-24}
12	66358347	T	C	0.054	1.60×10^{-17}
12	71500166	T	C	-0.046	9.30×10^{-13}
12	95927762	T	C	-0.035	5.30×10^{-08}
12	97846144	T	C	-0.071	3.10×10^{-07}
12	108618630	T	C	-0.049	1.40×10^{-11}
12	121416864	T	C	0.16	8.90×10^{-17}
12	123518866	T	C	-0.047	1.20×10^{-09}
12	124440110	A	C	-0.033	1.10×10^{-06}
12	124505444	T	C	0.032	1.50×10^{-06}
13	26781528	A	G	-0.044	7.00×10^{-09}
13	31037903	C	G	-0.041	2.00×10^{-08}
13	33554302	A	G	-0.053	6.80×10^{-10}
13	51094114	T	G	0.039	3.60×10^{-08}
13	80717156	A	G	-0.083	5.70×10^{-31}
13	110457029	A	G	-0.032	1.70×10^{-06}
14	23288953	A	G	-0.043	2.30×10^{-08}
14	33309186	T	C	-0.034	1.80×10^{-07}
14	38804433	A	G	-0.038	2.00×10^{-07}
14	79936964	T	C	-0.057	1.60×10^{-13}
14	103883633	T	G	-0.037	4.10×10^{-08}
15	38834033	T	C	-0.043	8.40×10^{-09}
15	53091870	A	G	0.047	2.70×10^{-07}
15	57535215	A	G	0.092	6.60×10^{-08}
15	62391608	T	C	-0.047	4.70×10^{-13}
15	63889022	A	G	-0.038	2.50×10^{-09}
15	68080886	A	T	0.039	6.20×10^{-09}
15	75927717	A	G	-0.043	6.60×10^{-09}
15	77747190	A	G	-0.078	9.20×10^{-29}

15	90373873	T	C	0.063	3.40×10^{-19}
16	295795	T	C	0.061	7.00×10^{-13}
16	28885659	T	C	0.029	5.80×10^{-06}
16	30018720	T	C	0.042	1.80×10^{-10}
16	53489705	A	G	-0.034	9.00×10^{-08}
16	53800954	T	C	-0.12	2.40×10^{-78}
16	69750849	T	C	-0.042	8.60×10^{-11}
16	75243142	T	C	-0.13	1.10×10^{-25}
16	81534790	T	C	0.053	2.10×10^{-14}
17	7549681	T	C	-0.028	1.30×10^{-05}
17	9788769	A	G	0.038	2.20×10^{-08}
17	17661802	A	G	0.048	3.90×10^{-12}
17	61965043	T	C	0.035	1.90×10^{-06}
17	65647063	A	G	-0.052	5.80×10^{-07}
17	65898809	A	G	0.052	4.40×10^{-11}
18	7076464	A	G	0.036	5.10×10^{-08}
18	53070168	T	C	0.081	1.20×10^{-12}
18	53430391	T	C	0.034	1.10×10^{-04}
18	56876228	A	G	-0.05	6.70×10^{-09}
18	58039276	T	C	-0.14	1.50×10^{-10}
18	60845884	T	C	0.049	5.10×10^{-13}
19	7235146	A	G	-0.045	2.40×10^{-08}
19	13010643	T	G	-0.043	1.10×10^{-10}
19	19407718	T	C	-0.091	1.50×10^{-14}
19	47569003	A	G	0.046	1.20×10^{-11}
20	21451139	A	G	0.057	1.30×10^{-07}
20	32698275	T	C	-0.042	6.90×10^{-10}
20	43042364	T	C	0.16	3.20×10^{-20}
20	45593210	A	G	-0.045	9.90×10^{-10}
20	57394420	T	C	0.043	2.70×10^{-11}

22	30592069	T	C	-0.081	1.60×10^{-12}
22	32484201	T	C	0.063	9.70×10^{-08}
22	41641984	T	C	-0.034	1.60×10^{-06}
22	44324727	C	G	-0.049	2.30×10^{-10}

GWAS data for the PPP-Botnia Study [22] (*n*=4928) genotyped using FinnGen ThermoFisher Axiom custom array was obtained from the FINNGEN Study (https://www.finngen.fi/en/researchers/genotyping). All participants in the *HNF1A* Study (*N*=145 carriers and 131 non-carriers) were genotyped at the genotyping unit of the Institute for Molecular Medicine Finland (FIMM), University of Helsinki using Illumina GlobalScreeninArray 24v3-0 (with ~730 000 markers) and customized Multi-Disease bead chip (with 34 1919 locally customized markers). The T2D PRS was based on T2D associated loci as reported by Mahajan et al [23]. We searched for these known T2D loci or their proxies (r^2>0.8 in the PPP-Botnia Study population) in the non-imputed genotype data, and calculated the PRS using LD pruning methods (r^2≥0.5) as implemented in Plink (version 1.9) using effect sizes reported by Mahajan et al. [23].

ESM Table 4. Characteristics of the carriers and non-carriers of HNF1A p.(Gly292fs) (mean adult values)

	Carriers			Non-carriers			Statistics			
Characteristic	Result	Age at investigation	*n* (% female)	Result	Age at investigation	*n* (% female)	MWU	95% CI	*p* value	LM est [a]
**HbA_{1c} *(mmol/mol)***	**53 [42–64]**	**43 [31–57]**	**113 (51%)**	**36 [33–40]**	**44 [31–58]**	**100 (43%)**	**15.6**	**12.2 ; 19.0**	**7.9×10^{-21}**	**18**
**(%)**	**7.0 [6.0-8.0]**			**5.4 [5.2-5.8]**						
**Fasting values[b]**										
**Glucose *(mmol/l)***	**7.7 [5.7–9.9]**	**43 [31–57]**	**115 (51%)**	**5.4 [5.1–5.8]**	**44 [32–59]**	**120 (46%)**	**1.9**	**1.3 ; 2.5**	**1.2×10^{-13}**	**2.4**
**Insulin *(pmol/l)***	**33 [22–50]**	**43 [31–59]**	**113 (51%)**	**41 [27–55]**	**44 [32–59]**	**118 (46%)**	**-7.0**	**-12.7 ; -1.3**	**0.015**	**-9.0**
**C-peptide *(nmol/l)***	**0.35 [0.24–0.46]**	**43 [31–58]**	**99 (51%)**	**0.44 [0.30–0.64]**	**43 [30–59]**	**97 (45%)**	**-0.1**	**-0.2 ; 0.0**	**0.0025**	**-0.12**
**Insulin:C-peptide**	**94 [69–126]**	**43 [31–59]**	**98 (50%)**	**110 [80–166]**	**43 [30–59]**	**96 (46%)**	**-19**	**-34 ; -4**	**0.013**	**-33** [c]
**Proinsulin *(pmol/l)***	**3.8 [2.8–7.3]**	**48 [31–62]**	**58 (55%)**	**6.7 [3.6–14.8]**	**53 [38–65]**	**45 (33%)**	**-2.4**	**-4.5 ; -0.7**	**0.0044**	**-5.9**
**OGTT glucose *(mmol/l)***										
**0 min**	**6.2 [5.3–7.5]**	**37 [29–51]**	**72 (54%)**	**5.4 [5.0–5.8]**	**43 [32–57]**	**107 (47%)**	**0.8**	**0.5 ; 1.2**	**1.5×10^{-6}**	**1.2**
**30 min**	**11.0 [9.4–13.2]**	**37 [29–52]**	**74 (54%)**	**8.4 [7.4–9.4]**	**43 [32–57]**	**108 (46%)**	**2.4**	**1.8 ; 3.1**	**2.6×10^{-12}**	**2.8**
**60 min**	**13.6 [11.1–16.4]**	**37 [30–52]**	**74 (54%)**	**7.9 [6.3–9.9]**	**43 [32–57]**	**108 (46%)**	**5.5**	**4.5 ; 6.6**	**1.0×10^{-16}**	**5.7**
**90 min**	**15.0 [10.8–18.3]**	**43 [32–53]**	**59 (54%)**	**6.5 [5.1–8.2]**	**45 [32–59]**	**75 (47%)**	**7.40**	**5.7 ; 9.2**	**1.0×10^{-14}**	**7.7**
**120 min**	**12.6 [9.3–16.8]**	**37 [29–51]**	**72 (54%)**	**6.0 [5.2–7.5]**	**43 [32–57]**	**107 (47%)**	**6.1**	**4.9 ; 7.8**	**9.6×10^{-19}**	**7.1**
**OGTT insulin *(pmol/l)***										
**0 min**	**29 [20–40]**	**37 [29–52]**	**70 (53%)**	**41 [27–55]**	**44 [32–57]**	**106 (47%)**	**-11**	**-17 ; -5**	**0.00026**	**-15**
**30 min**	**110 [68–153]**	**37 [29–52]**	**70 (53%)**	**328 [221–503]**	**43 [32–57]**	**107 (47%)**	**-215**	**-267 ; -169**	**2.8×10^{-19}**	**-243**
**60 min**	**127 [88–175]**	**37 [29–52]**	**70 (53%)**	**364 [217–554]**	**43 [32–57]**	**107 (47%)**	**-230**	**-285 ; -175**	**1.8×10^{-17}**	**-285**
**90 min**	**133 [92–219]**	**45 [32–56]**	**58 (52%)**	**346 [209–520]**	**45 [32–59]**	**75 (47%)**	**-202**	**-266 ; -136**	**6.0×10^{-10}**	**-278**
**120 min**	**111 [62–171]**	**37 [29–52]**	**70 (53%)**	**230 [130–393]**	**43 [32–57]**	**106 (47%)**	**-118**	**-163 ; -77**	**4.2×10^{-9}**	**-188**
**OGTT C-peptide *(nmol/l)***										
**0 min**	**0.35 [0.24–0.46]**	**38 [30–52]**	**71 (54%)**	**0.45 [0.31–0.65]**	**43 [30–59]**	**92 (48%)**	**-0.1**	**-0.2 ; 0.0**	**0.0049**	**-0.12**

**30 min**	**0.79 [0.47–0.99]**	**38 [31–52]**	**69 (54%)**	**1.59 [1.05–2.00]**	**43 [30–59]**	**93 (47%)**	**-0.78**	**-0.9 ; -0.6**	**2.0×10^{-11}**	**-0.73**
**120 min**	**1.03 [0.62–1.52]**	**38 [30–52]**	**71 (54%)**	**1.7 [1.03–2.44]**	**43 [30–59]**	**92 (48%)**	**-0.6**	**-0.8 ; -0.3**	**3.0×10^{-5}**	**-0.65**
**OGTT proinsulin *(pmol/l)***										
**0 min**	**3.7 [2.4–5.8]**	**46 [30–58]**	**41 (49%)**	**6.7 [3.7–14.6]**	**54 [38–66]**	**43 (35%)**	**-3.0**	**-5.1 ; -1.3**	**0.0003**	**-5.3**
**120 min**	**13.9 [9.9–24.2]**	**46 [30–58]**	**41 (49%)**	**41.4 [27.8–73.8]**	**54 [38–66]**	**43 (35%)**	**-24.7**	**-35.8 ; -16.9**	**1.2×10^{-9}**	**-37**
**OGTT insulin:C-peptide**										
**30 min**	**152 [120–186]**	**38 [30–52]**	**67 (54%)**	**251 [197–371]**	**43 [30–59]**	**92 (48%)**	**-102**	**-133 ; -74**	**5.5e-^{11}**	**(-229)**
**120 min**	**113 [80–150]**	**38 [30–52]**	**69 (54%)**	**137 [107–292]**	**44 [31–59]**	**91 (48%)**	**-39**	**-67 ; -18**	**0.00017**	**(-146)**
**Indices of glucose metabolism**										
**CIR** [d]	**19 [10–43]**	**38 [29–52]**	**69 (54%)**	**131 [80–186]**	**43 [32–57]**	**106 (46%)**	**-103**	**-122 ; -86**	**1.2×10^{-20}**	**-119** [c]
**HOMA2β (%)**	**35 [22–55]**	**43 [30–59]**	**108 (52%)**	**71 [55–86]**	**44 [32–60]**	**117 (45%)**	**-33.7**	**-40 ; -27.3**	**9.9×10^{-17}**	**-35**
HOMA2 IS (%)	133 [91–203]	43 [30–59]	108 (52%)	123 [86–188]	44 [32–60]	117 (45%)	12.2	-8.1 ; 32.8	0.24	-450
**ISI** [d]	**8.9 [5.7–13.1]**	**37 [29–51]**	**66 (55%)**	**6.1 [4–9.3]**	**44 [32–57]**	**105 (47%)**	**2.51**	**1.2 ; 3.8**	**0.00023**	**2.6**
**Lipid and fatty acid metabolism, creatinine**										
Total cholesterol	4.8 [4.3–5.3]	41 [30–57]	117 (52%)	4.9 [4.3–5.5]	44 [32–59]	119 (45%)	-0.2	-0.4 ; 0.1	0.14	-0.16
**HDL cholesterol**	**1.5 [1.2–1.7]**	**42 [30–57]**	**116 (53%)**	**1.3 [1.1–1.5]**	**44 [32–59]**	**119 (45%)**	**0.1**	**0.1 ; 0.2**	**0.0012**	**0.13**
Triglyserides	1.1 [0.8–1.4]	41 [30–57]	117 (52%)	1 [0.8–1.5]	44 [32–59]	118 (45%)	0.0	-0.1 ; 0.1	0.94	-0.022
LDL cholesterol	2.5 [2.0–3.0]	49 [30–62]	77 (56%)	2.8 [2.3–3.2]	53 [35–64]	57 (39%)	-0.2	-0.5 ; 0.1	0.16	-0.17
**Non-HDL cholesterol**	**3.4 [2.6–3.8]**	**42 [30–57]**	**116 (53%)**	**3.6 [3–4.3]**	**44 [32–59]**	**119 (45%)**	**-0.3**	**-0.6 ; -0.1**	**0.014**	**-0.28**
**Fasting NEFA**	**621 [471–820]**	**48 [32–63]**	**64 (56%)**	**459 [340–648]**	**53 [35–64]**	**50 (36%)**	**151.8**	**51.8 ; 243.4**	**0.0027**	**120**
0-min NEFA (OGTT)	588 [387–766]	45 [30–59]	46 (50%)	439 [335–643]	53 [36–64]	47 (38%)	109.3	6.7 ; 214.9	0.042	120
**120-min NEFA**	**115 [79–177]**	**45 [30–59]**	**46 (50%)**	**64 [45–91]**	**53 [36–64]**	**47 (38%)**	**49.3**	**25.5 ; 73.2**	**4.3×10^{-5}**	**110**
Creatinine	76 [66–87]	49 [33–60]	97 (52%)	79 [68–90]	45 [32–62]	99 (43%)	-3.0	-8.0 ; 1.0	0.15	-0.6
GFR (by CKD-EPI)	93 [80–106]	44 [33–59]	107 (51%)	91 [77–105]	43 [32–60]	102 (43%)	2.0	-3.6 ; 7.5	0.46	0.88
**Anthropometric measurements**										
Weight *kg*	74 [63–84]	49 [33–62]	107 (52%)	77 [66–91]	51 [35–64]	117 (46%)	-4.4	-9.0 ; 0.1	0.059	-4.4
Height *cm*	170 [164–178]	43 [31–57]	107 (52%)	170 [163–178]	44 [32–60]	117 (46%)	0.4	-2.3 ; 3.2	0.77	0.92
Waist circumference *cm*	90 [81–98]	50 [33–62]	104 (53%)	91 [84–105]	51 [35–65]	116 (46%)	-3.0	-7.5 ; 1.0	0.11	-2.4
**BMI kg/m^2**	**24 [22–27]**	**43 [31–57]**	**107 (52%)**	**26 [23–30]**	**44 [32–60]**	**117 (46%)**	**-2.0**	**-3.2 ; -0.9**	**0.00052**	**-1.8**

Body fat percentage (%)	27 [22–32]	50 [33–62]	61 (54%)	30 [23–37]	53 [37–65]	47 (38%)	-2.5	-6.5 ; 1.3	0.2	-3.5
Lean body mass *kg*	52 [45–63]	49 [33–62]	99 (55%)	55 [45–65]	52 [35–64]	110 (45%)	-1.9	-5.4 ; 1.3	0.24	-0.72
**Fat body mass *kg***	**19 [14–26]**	**49 [33–62]**	**99 (55%)**	**22 [17–28]**	**52 [35–64]**	**110 (45%)**	**-3.0**	**-5.3 ; -0.5**	**0.016**	**-3.2**
Systolic blood pressure *mmHg*	128 [118–140]	43 [31–57]	107 (52%)	128 [118–140]	44 [32–60]	117 (46%)	0.0	-4.8 ; 4.5	1	1.1
Diastolic blood pressure *mmHg*	77 [71–83]	43 [31–57]	107 (52%)	79 [72–84]	44 [32–60]	117 (46%)	-1.1	-3.5 ; 1.2	0.31	-0.75

Mean adult levels of glucose, insulin, C-peptide, proinsulin and lipids at fasting and during an OGTT besides anthropometric measurements, and the mean age at assessment. The fasting levels are shown separately for the whole group and for the subgroup who participated in an OGTT (see methods). In *t*-testing, both FICR and CIR lost their statistical significance but were not normally distributed. *N* (% female), total number of individuals (the proportion of women); MWU, Estimator of the Mann-Whitney *U* test; CI, 95% confidence interval; LM est, linear model estimate; CIR, corrected insulin response; ISI, composite insulin sensitivity index (Matsuda index)

[a] Linear model estimate adjusted for age and sex.

[b] Including the 0 min values reported for the OGTT.

[c] One outlier was excluded for CIR and ten for fasting insulin:C-peptide ratio.

[d] CIR (at 30 min) in 100 pmol/l $(mmol/l)^{-2}$, ISI in 10 000 $(mg/dl)^{-1}$ $(mU/l)^{-1}$

ESM Table 5. Interaction model of glucose responsiveness

Response variable	Time point (during the OGTT, *min*)	Model intercept	*p* for the intercept	Estimate for the carrier status (as compared to non-carriers)	*p* for the carrier status	Estimate for plasma glucose	*p* for plasma glucose	Interaction term between carrier status and plasma glucose	*p* for the interaction	R^2 adjusted
**S- Insulin**	**0** [a]	**-30.1**	**3.1×10^{-3}**	**43.8**	**3.9×10^{-4}**	**12.5**	**7.6×10^{-12}**	**-9.78**	**6.4×10^{-7}**	**0.22**
**S- Insulin**	**30**	**-89.4**	**0.30**	**284**	**0.025**	**50.7**	**9.3×10^{-7}**	**-58.3**	**1.1×10^{-5}**	**0.34**
**S- Insulin**	**60**	**-104**	**0.21**	**296**	**0.035**	**63.2**	**7.3×10^{-10}**	**-67.9**	**2.4×10^{-7}**	**0.32**
**S- Insulin**	**90**	**-285**	**4.6×10^{-4}**	**484**	**5.4×10^{-5}**	**107**	**5.7×10^{-17}**	**-110**	**4.5×10^{-15}**	**0.52**
**S- Insulin**	**120**	**-96.7**	**0.14**	**286**	**3.7×10^{-3}**	**61.0**	**2.8×10^{-9}**	**-66.0**	**1.4×10^{-8}**	**0.25**
S- C-peptide	0 [a]	0.39	1.1×10^{-5}	0.072	0.53	0.0008	0.96	-0.018	0.31	0.017
S- C-peptide	30	1.10	1.8×10^{-3}	0.11	0.83	0.025	0.53	-0.067	0.18	0.14
S- C-peptide	120	1.17	6.5×10^{-5}	0.45	0.27	0.051	0.23	-0.089	0.058	0.048

Estimates and *p* values for coefficients from linear models using the carrier status, plasma glucose level and their interaction term as predictors. The models with a significant interaction are in bold.

[a] Including all fasting samples.

ESM Table 6. Characteristics of the carriers and non-carriers of HNF1A p.(Gly292fs) not shown in Table 1

	Carriers			Non-carriers			Statistics			
	Result	Age at investigation	*N* (% female)	Result	Age at investigation	*N* (% female)	MWU	CI	*p* value	LM est[a]
**Lipid and fatty acid metabolism, creatinine**										
Total cholesterol	4.9 [4.1–5.3]	38 [29–54]	117 (52%)	5 [4.3–5.7]	43 [31–55]	119 (45%)	-0.2	-0.5 ; 0.1	0.15	-0.13
**HDL cholesterol**	**1.4 [1.2–1.7]**	**38 [29–54]**	**116 (53%)**	**1.3 [1.1–1.6]**	**43 [31–55]**	**119 (45%)**	**0.1**	**0.0 ; 0.2**	**0.0094**	**0.10**
Triglyserides	1.0 [0.8–1.4]	38 [29–54]	117 (52%)	1.0 [0.7–1.4]	42 [31–55]	118 (45%)	0.0	-0.1 ; 0.1	0.61	0.005
LDL cholesterol	2.6 [2–3.1]	46 [30–62]	77 (56%)	2.8 [2.3–3.3]	53 [33–63]	57 (39%)	-0.2	-0.6 ; 0.1	0.14	-0.19
Non-HDL cholesterol	3.4 [2.5–4]	38 [29–54]	116 (53%)	3.7 [2.9–4.4]	43 [31–55]	119 (45%)	-0.3	-0.6 ; 0	0.037	-0.22
**Fasting NEFA *(μmol/l)***	**621 [452–829]**	**47 [32–63]**	**64 (56%)**	**441 [340–648]**	**53 [35–62]**	**50 (36%)**	**152.5**	**47.5 ; 252.5**	**0.0039**	**130**
0-min NEFA (OGTT)	576 [387–766]	45 [30–59]	46 (50%)	429 [335–643]	53 [35–63]	47 (38%)	108.5	3.4 ; 214	0.043	120
**120-min NEFA**	**117 [80–177]**	**45 [30–59]**	**46 (50%)**	**64 [45–91]**	**53 [35–63]**	**47 (38%)**	**50.1**	**26.9 ; 74.3**	**3.1×10^{-5}**	**110**
Fasting apo-CIII [b]	10.5 [8.5–13.0]	48 [30–59]	41 (61%)	9.5 [7.7–11.2]	53 [38–57]	27 (30%)	1.1	-0.3 ; 2.5	0.14	1.7
120-min apo-CIII	13.0 [9.9–14.8]	43 [29–56]	20 (60%)	11.2 [8.9–12.0]	54 [39–58]	21 (33%)	1.5	-0.8 ; 3.5	0.12	2.5
Creatinine *(μmol/l)*	81 [69–91]	39 [30–56]	97 (52%)	83 [73–94]	41 [32–54]	99 (43%)	-3.0	-8 ; 1	0.16	-0.47
GFR (by CKD-EPI)	91 [78–108]	40 [30–56]	107 (51%)	91 [77–104]	40 [32–54]	102 (43%)	1.6	-4.1 ; 7.2	0.57	0.62
**Anthropometric measurements**										
**Weight *(kg)***	**71 [60–78]**	**38 [29–53]**	**107 (52%)**	**75 [64–88]**	**43 [31–55]**	**117 (46%)**	**-5.0**	**-9.1 ; -1**	**0.012**	**-4.6**
Height *(cm)*	170 [165–178]	38 [29–53]	107 (52%)	171 [163–178]	43 [31–55]	117 (46%)	0.0	-2.5 ; 3	0.82	0.82
Waist circumference *(cm)*	86 [76–92]	38 [29–52]	104 (53%)	89 [80–100]	43 [32–55]	116 (46%)	-4.0	-8 ; -0.5	0.028	-2.4
**BMI *(kg/m^2)***	**23.5 [21.1–26.1]**	**38 [29–53]**	**107 (52%)**	**26.0 [22.8–28.9]**	**43 [31–55]**	**117 (46%)**	**-2.1**	**-3.1 ; -1.0**	**0.00022**	**-1.8**
Body fat percentage (%)	27 [22–32]	49 [33–62]	61 (54%)	30 [23–37]	53 [37–65]	47 (38%)	-2.6	-6.5 ; 1.2	0.19	-3.5
Lean body mass *(kg)*	50.5 [44.7–61.4]	39 [29–53]	99 (55%)	55.4 [45.6–65.1]	43 [32–55]	110 (45%)	-2.6	-6 ; 0.6	0.11	-1.3
**Fat body mass *(kg)***	**16.9 [12.6–22.4]**	**39 [29–53]**	**99 (55%)**	**19.7 [16.2–26.0]**	**43 [32–55]**	**110 (45%)**	**-3.0**	**-5.0 ; -1.1**	**0.0022**	**-2.9**

Systolic blood pressure *(mmHg)*	127 [113–138]	38 [29–53]	107 (52%)	128 [118–140]	43 [32–55]	117 (46%)	-1.0	-6.0 ; 3.5	0.62	0.056
Diastolic blood pressure *(mmHg)*	77 [69–83]	38 [29–53]	107 (52%)	79 [72–84]	43 [32–55]	117 (46%)	-2.0	-4.0 ; 1.0	0.19	-1.3

First adult levels (median [interquartile range]) of the data used for the multiple hypothesis correction by Benjamini-Hochberg procedure and partially shown in Table 1. Only the results in bold remained significant. Cholesterol and triglyseride values in *mmol/l.* Of note, the difference between carriers' and non-carriers' fat mody mass was significant only in men (-4.2 kg [CI -6.8 ; -1.3], *p*=0.0042) but not in women (-2.2 kg [-4.8 ; 0.6], *p*=0.13). *N* (% female), total number of individuals (the proportion of women); MWU, estimator of Mann-Whitney *U* test; CI, 95% confidence interval; LM est, linear model estimate; GFR, glomerular filtration rate.

[a] Linear model estimate adjusted for age and sex.

[b] Including the 0 min values reported for the OGTT

ESM Fig. 1

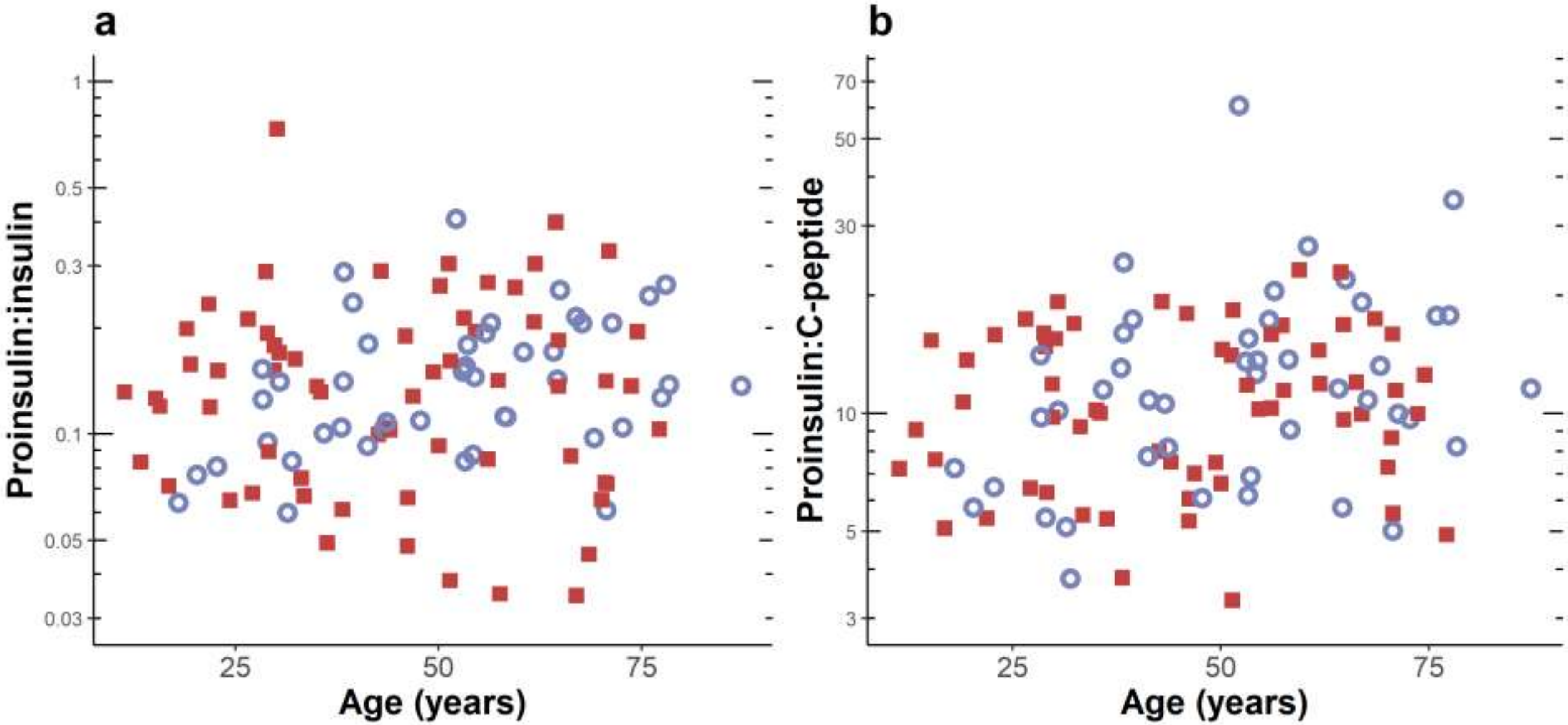

ESM Fig. 1 illustrates the ratio of proinsulin to insulin (panel A) or C-peptide (panel B) at fasting (y- axis, log scale) according to age (x- axis) in carriers (red square boxes) and non-carriers (blue circles).

ESM Fig. 2

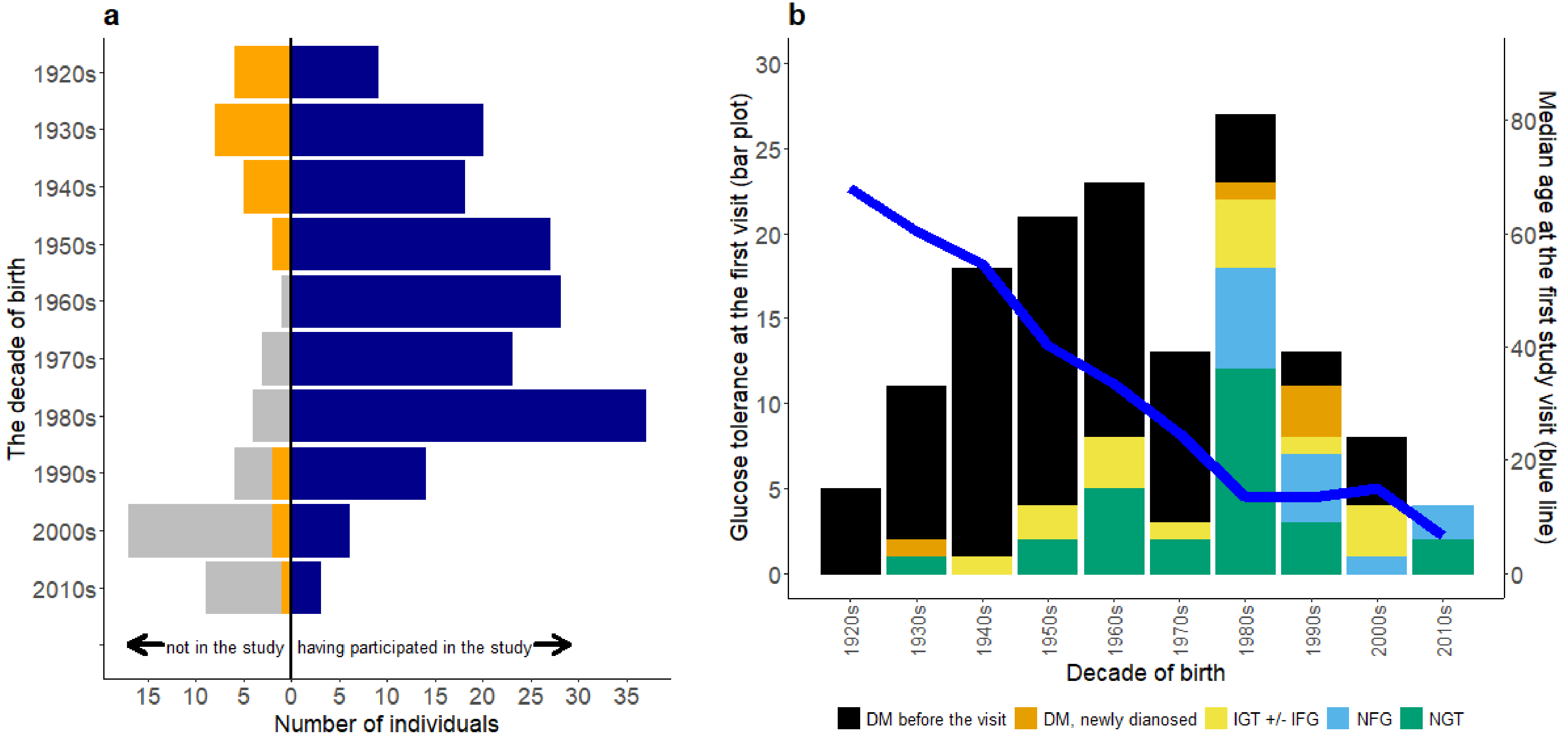

ESM Fig. 2. Panel A shows the number (x-axis) of the participants (blue bars) and their not participating siblings (orange bars) from the three large families B, C and D stratified by the decade of birth (yaxis). The grey bars represent the carriers' offspring if none of a carrier's children have participated. Panel B shows the number of all carriers in this study (y-axis on the left) according to their initial glucose tolerance (as specified in the figure, bar plot) as well as the median age at the first visit (blue line, y-axis on the right) stratified by the decade of birth (x-axis).

ESM Fig. 3

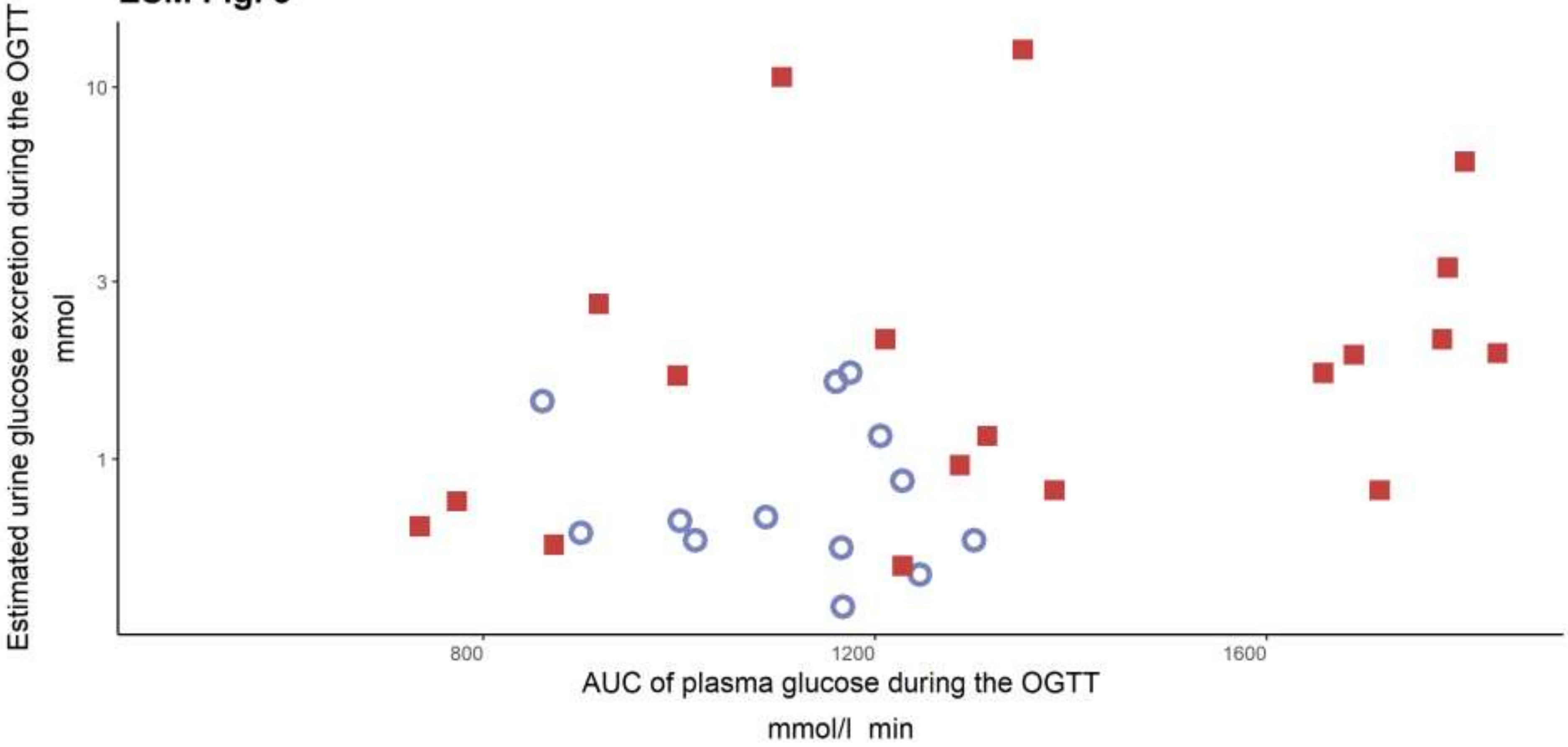

ESM Fig. 3. The area under the curve (AUC) of plasma glucose during OGTT (x-axis) plotted against glucose excreted in urine during the OGTT (y-axis) in those carriers (red square boxes) and non-carriers (blue circles) of the *HNF1A* p.(Gly292fs) variant, whose urine sample before the OGTT was negative for glucose in a semiquantative dipstick analysis. Using an estimate of glucose concentration by a dipstick analysis after the OGTT, the urine glucose is calculated as $OGTT\ urine\ volume\ (ml) \cdot semiquantitative\ urine\ concentration\ (\frac{mmol}{ml})$.

ESM References

ESM1. Stride A, Ellard S, Clark P, et al (2005) Beta-cell dysfunction, insulin sensitivity, and glycosuria precede diabetes in hepatocyte nuclear factor-1alpha mutation carriers. Diabetes Care 28(7):1751–1756. https://doi.org/10.2337/diacare.28.7.1751

All other references according to the main text.
